# Therapeutic antibodies under development for SARS‐CoV‐2

**DOI:** 10.1002/VIW.20200178

**Published:** 2021-07-12

**Authors:** Zetong Ma, MengMei Zhu, Shuyi Zhang, Kewen Qian, Chuqi Wang, Wenyan Fu, Changhai Lei, Shi Hu

**Affiliations:** ^1^ Department of Biophysics College of Basic Medical Sciences Second Military Medical University Shanghai China; ^2^ Team SMMU‐China of the International Genetically Engineered Machine (iGEM) competition Department of Biophysics Second Military Medical University Shanghai China; ^3^ Department of Assisted Reproduction Shanghai Ninth People's Hospital Shanghai Jiao Tong University School of Medicine Shanghai China

**Keywords:** Ig‐like proteins, neutralization, SARS‐CoV‐2, SARS‐CoV

## Abstract

The world is experiencing one of the most difficult moments in history with COVID‐19, which has rapidly developed into a worldwide pandemic with a significant health and economic burden. Efforts to fight the virus, including prevention and treatment, have never stopped. However, no specific drugs or treatments have yet been found. Antibody drugs have never been absent in epidemics such as SARS, MERS, HIV, Ebola, and so on in the past two decades. At present, while research on the SARS‐CoV‐2 vaccine is in full swing, antibody drugs are also receiving widespread attention. Several antibody drugs have successfully entered clinical trials and achieved impressive therapeutic effects. Here, we summarize the therapeutic antibodies against SARS‐CoV‐2, as well as the research using ACE2 recombinant protein or ACE2‐Ig fusion protein.

## Graphical abstract

Several antibody drugs have successfully entered clinical trials and achieved impressive therapeutic effects. Due to the rapid progress in the field of therapeutic antibodies for COVID‐19 treatment, the authors summarize the neutralizing antibody drugs and fusion protein drugs that have attracted high attention at present, discuss the existing problems in the current application of antibodies drugs for COVID‐19, and prospect the future research direction in this field.

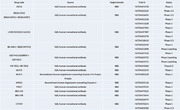

**Abbreviations**
ACE2: angiotensin‐converting enzyme 2
ADE: antibody‐dependent enhancement
ARDS: acute respiratory distress syndrome
COVID‐19: Corona Virus Disease 2019
CP: convalescent plasma
HIV: human immunodeficiency virus
MERS: Middle East respiratory syndrome
Nabs: neutralizing antibodies
NRSIs: non‐randomized studies of the effects of interventions
NTD: N‐terminal domain
RBD: receptor‐binding domain
RCT: randomized controlled trial
RT‐QPCR: reverse‐transcribed quantitative polymerase chain reaction
SARS: severe acute respiratory syndrome

## 1 INTRODUCTION

In late 2019, a novel coronavirus emerged in the world and caused a widespread acute respiratory disease, which is known as coronavirus disease 2019 (COVID‐19).^[^ ^1^ ^]^ Severe acute respiratory syndrome coronavirus 2 (SARS‐CoV‐2) that falls in the category of novel β‐coronavirus, formerly known as the 2019 novel coronavirus (2019‐nCoV), is the causative agent of this global health threat.^[^ ^2^ ^]^ SARS‐CoV‐2 enters into host cells following a multi‐step process. The surface of SARS‐CoV‐2 is covered with S protein, which comprises two functional subunits responsible for binding to the host cell receptor (S1 subunit) and fusion of the viral and cellular membranes (S2 subunit). S1 is subdivided in domains S1A (or NTD) and S1B (or RBD), the latter binding to the ACE2 receptor.^[^ ^3^ ^]^ Large‐scale conformational changes of the S protein facilitate the fusion process of virus‐host cell membrane, thereby initiating host cell entry.^[^ ^4^ ^]^

**FIGURE 1:**
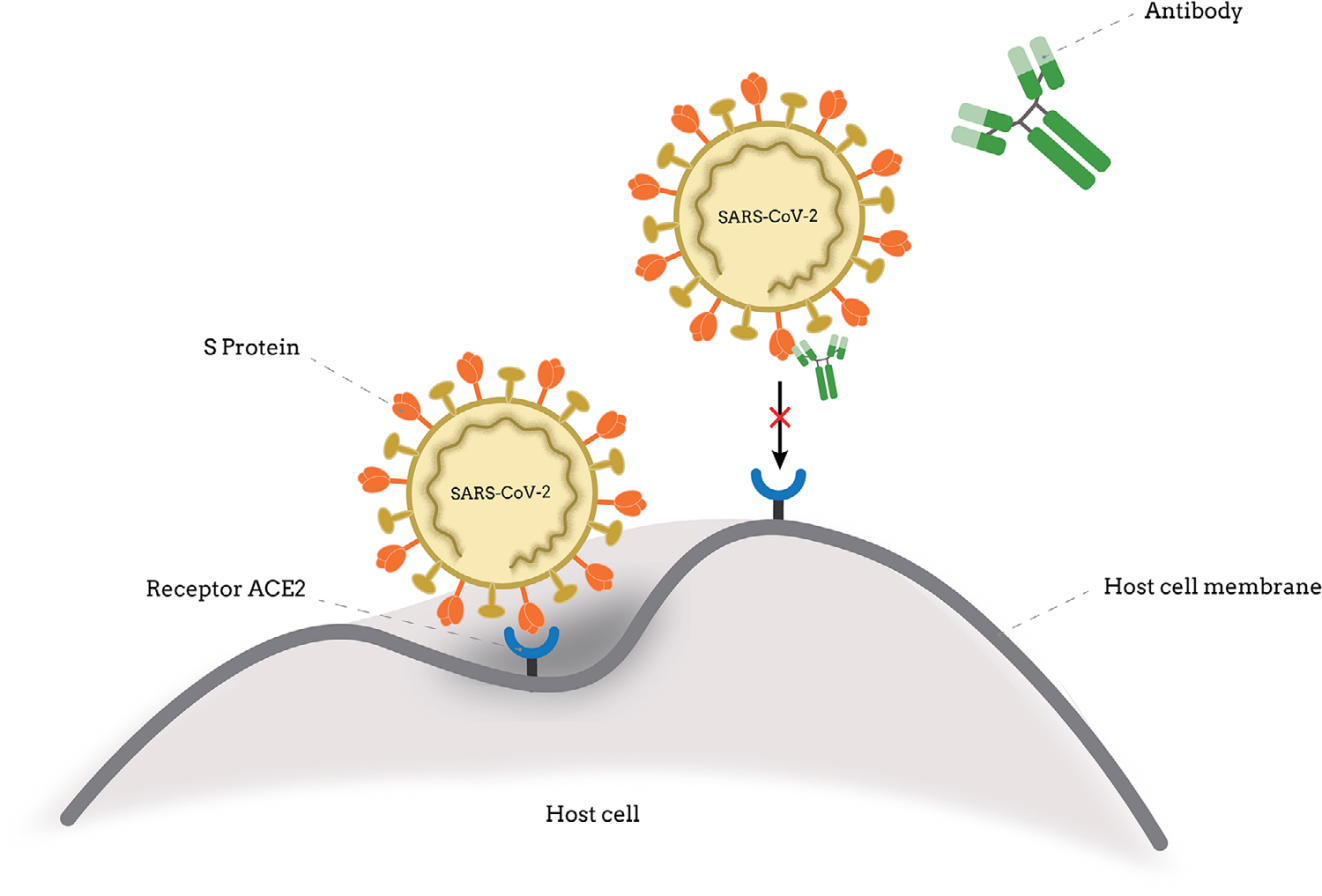
Antibodies work to neutralize viruses by binding to their surface and blocking entry into host cells

SARS‐CoV‐2 recognizes ACE2 the same as SARS‐CoV and coronavirus NL63 (HCoV‐NL63).^[^ ^5^ ^]^ Expressions of ACE2 receptors are detected in the lungs, cardiovascular system, central nervous intestine, kidney, system, and adipose tissue. ACE2 receptors mediate SARS‐CoV‐2 into cells, suggesting a critical link between ACE2, immune system, inflammation, and cardiovascular disease.^[^ ^6^ ^]^ Comparing with the receptor‐binding domain (RBD) of SARS‐CoV, several residual changes in the SARS‐CoV‐2 RBD increase its ACE2 binding affinity.^[^ ^7^ ^]^ What's more, the antigenicity of SARS‐CoV and SARS‐CoV‐2 is significantly different.^[^ ^8^ ^]^ COVID‐19 spreads quickly worldwide and causes diseases of varying degrees. Although most infected people only have mild illnesses, about 15–20% of people develop serious illnesses that require hospitalization, while 5% require admission to the intensive care unit. Typical COVID‐19 symptoms and potential atypical symptoms assessed included fever, cough, shortness of breath, increased confusion, headache, muscle ache, malaise, sore throat, diarrhea, chest pain, rhinorrhea or nasal congestion, and nausea and vomiting.^[^ ^1^ ^]^ From the imaging examinations, only a small number of patients found no imaging or CT abnormalities.^[^ ^9^ ^]^ However, there are currently no specific treatments for COVID‐19. To date, many potential therapeutic strategies have been revealed based on the research progress of SARS‐CoV‐2,^[^ ^10^ ^]^ including blocking SARS‐CoV‐2 fusion/entry, disrupting SARS‐CoV‐2 replication, suppressing excessive inflammation response, convalescent plasma treatment, vaccines^[^ ^11^ ^]^ as well as the combination of Traditional Chinese and Western medicine.^[^ ^12^ ^]^ Additionally, a number of clinical trials are ongoing to test the effectiveness and safety of candidate drugs, including glucocorticoids, JAK inhibitors, IL‐6 antagonists, chloroquine/hydroxychloroquine, and convalescent plasma.^[^ ^13^ ^]^ Preliminary trials for convalescent plasma indicate that there may be some benefits.^[^ ^14^ ^]^ However, convalescent plasma as a treatment for COVID‐19 still has high risks, and randomized controlled trials should be conducted promptly to determine its efficacy and biosafety.^[^ ^15^ ^]^ Therapeutic antibodies have been used in the treatment of large‐scale infectious diseases and have achieved certain therapeutic effects. The discovery and development of virus‐neutralizing monoclonal antibodies could be a way to treat or prevent this coronavirus infection. The spike glycoprotein has attracted considerable attention due to its key role in SARS‐CoV‐2 cell entry mechanism. Disrupting its interaction with the ACE2 receptor has been used as a potential intervention strategy for viral cell entry.^[^ ^16^ ^]^ SARS‐CoV‐2 neutralizing monoclonal antibodies isolated from patients infected with SARS‐CoV‐2 and hospitalized with COVID‐19 found that almost all antibodies that can neutralize SARS‐CoV‐2 are directed against the S protein receptor binding structure domain (RBD) and the n‐terminal domain (NTD), indicating that both of these regions at the top of the viral spike are immunogenic and making monoclonal antibodies promising as potential drugs for the treatment and/or prevention of SARS‐CoV‐2 for clinical development (Figure 1).^[^ ^17^ ^]^ In addition, as a single antibody may be insufficient and could lead to mutations, the prospect of using a mixture of antibodies has been put forward.^[^ ^18^ ^]^

In this review, we summarize the current knowledge about SARS‐CoV‐2 antibody drugs therapy based on emerging basic research and clinical data.

## 2 NEUTRALIZING SARS‐COV‐2 THROUGH A RECEPTOR ANTAGONISM MECHANISM

### 2.1 CA1 CB6

2.1

**FIGURE 2:**
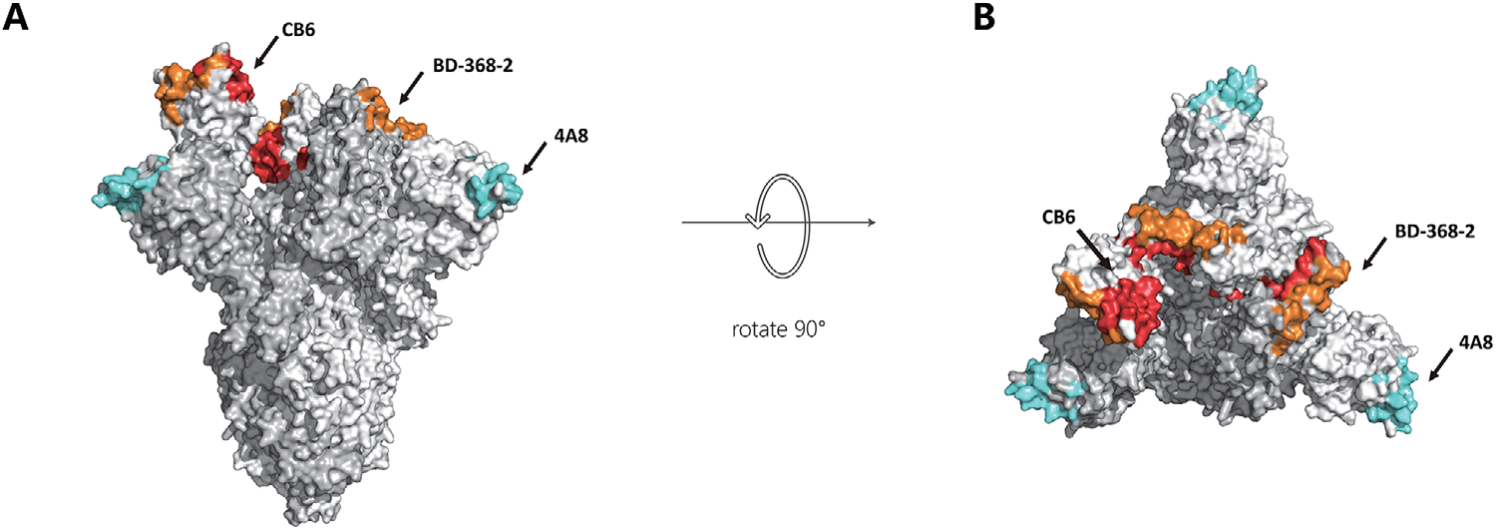
The epitope of some therapeutic antibodies for COVID‐19. (A)The side view of the SARS‐CoV‐2 S trimer)PDB ID:7DK3(. (B)The top view of the SARS‐CoV‐2 S trimer. The spike protein trimer is shown in white, CB6 binding site in red, BD‐368‐2 in orange, 4A8 in cyan

Shi R et al. reported two specific human monoclonal antibodies (CA1 and CB6) isolated from a COVID‐19 convalescent plasma. They tested and identified the neutralization ability using pseudoviruses and living viruses in vitro. These two monoclonal antibodies showed significant neutralizing activity against SARS‐CoV‐2, while CB6 showed stronger than CA1. CB6 decrease the virus titer and reduced infection‐related lung injury in rhesus monkey models, whether for prevention or treatment. The CB6 binding epitopes on RBD overlaps with ACE2 recognition epitopes, indicating that CB6 could block the SARS‐CoV‐2 recognize ACE2 receptor and prevent it from entering cells (Figure 2). At the same time, the overlapped epitopes also ensured that antibodies could effectively avoid virus generate mutation that may result in drug resistance. The team modified the Fc segment of the CB6 to introduce LALA mutations to reduce its mediated cytotoxic effects and reduce the risk of acute lung injury. In animal experiments, both the treatment group and the prevention group had intact alveolar structures and showed limited pathological lung damage, indicating that the engineered CB6‐LALA antibody significantly inhibited the SARS‐CoV‐2 virus infection while reducing infection‐related lung damage.^[^ ^19^ ^]^

### 2.2 REGN10987, REGN10933

Regeneron screened out more than 200 candidate antibodies initially based on the transgenic mouse model and blood of COVID‐19 recovered patients. Finally, the two most suitable monoclonal antibodies were selected to form cocktail therapy according to the binding ability, neutralization ability, and three‐dimensional structure. REGN10987 and REGN10933 are non‐competitive and simultaneously bind to RBD, blocking the binding interface between RBD and human ACE2. REGN10933 targets the spike‐shaped loop region on one edge of the ACE2 interface and binds virus RBD from the top, which greatly hinds the contract between virus RBD and host cells, while REGN10987 is located on the side of the RBD and can only bind to the RBD from the front of the lower left side. It is separated from the REGN10933 epitope, and barely overlapped with the ACE2 binding site.

In Phase 1/2/3 clinical trials (NCT04425629), 275 patients who were randomly distributed in the proportion 1:1:1 were each given a one‐time infusion of 2.4 g of REGN‐COV2 (low dose), 8 g of REGN‐COV2 (high dose), or placebo. The participants were divided into two groups based on the SARS‐COV‐2 antibody seropositive or seronegative. The seropositive patients had lower baseline virus levels, and their viral load could quickly drop to the lowest quantifiable levels without treatment. In the placebo treatment group, the median time to remission for seropositive patients was 7 days, while the median time to remission for seronegative patients was 13 days. On day 7 of REGN‐COV2 treatment, the viral load of patients with a higher virus baseline had a corresponding reduction. Patients with serum negative and/or higher virus baseline levels benefited more in terms of symptom alleviation.^[^ ^20^ ^]^

### 2.3 BD‐368‐2

Beigene and Singlomics collaborated to develop a series of SARS‐CoV‐2 neutralizing antibodies from the antigen‐binding B cells of COVID‐19 convalescents by single‐cell RNA and VDJ sequencing. Among them, BD‐368‐2 has the best neutralization property against the pseudotyped and authentic virus. In vivo experiments using the human ACE2 transgenic mouse model shows that given BD‐368‐2 before the viral challenge could prevent SARS‐CoV‐2 infection completely while giving BD‐368‐2 2 h after infection decreased virus load in mice lung rapidly, proved that BD368‐2 has strong protective and preventive effects. The 3.5‐Å cryo‐EM structure shows that BD‐368‐2 can completely cover all three RBDS (Figure 2), completely change the conformation of spike protein trimer, and thus completely block the virus recognition of ACE2.^[^ ^21^ ^]^

BD‐368‐2, also called BGB‐DXP593, is in phase II study with ongoing randomized, double‐blind, and placebo‐controlled trials (NCT04551898). The trial recruited about 180 patients aged 18‐65 years who developed symptoms of COVID‐19 (such as fever, sore throat, cough, vomiting, diarrhea, visual impairment, and shortness of breath) for 7 days. Participants were assigned to one of three treatment groups at different doses and a control group. All participants received treatment and were followed up safely for 85 days. This trial evaluates the safety and tolerability of BGB‐DXP593 in single‐dose intravenous administration on patients who have mild or moderate symptoms of COVID‐19. The primary outcome will assess the changes in virus spread from baseline to day 8, measured by RT‐QPCR (reverse transcribed quantitative polymerase chain reaction) in nasopharyngeal swabs.

### 2.4 AZD7442)COV2‐2196 and COV2‐2130(

Zost SJ et al. obtained 389 SARS‐CoV‐2 S‐protein‐reactive monoclonal antibodies from two SARS‐CoV‐2 infected patients who were recovering and tested their potency against SARS‐COV‐2 neutralizing and protective human antibodies.^[^ ^22^ ^]^ Researchers found that these neutralizing monoclonal antibodies can effectively identify non‐overlapping sites and bind to the S protein simultaneously to synergistically eliminate SARS‐CoV‐2 virus. They determined that the two monoclonal antibodies, COV2‐2196 and COV2‐2130, had the most powerful blocking activity against S protein, also had the highest level of neutralization efficiency and excellent binding activity to S trimer. The binding of human ACE2 was completely blocked and the S trimer was bound firmly by both of them. COV2‐2130 recognizes the two configurations of the RBD when the S trimer is in close or open position, and binds the Fab simultaneously. COV2‐2196 and COV2‐2165 can bind to RBD when the S protein trimer is in the “OPEN” position. Researchers used mutagenesis to confirm the key binding residues in the RBD. The study revealed that N487 and F486 are pivotal residues that bind with COV2‐2196, while N487 is a pivotal residue for binding with COV2‐2165—two antibodies compete with each other in the binding process. Similarly, K444A and G447R mutants are essential for COV2‐2130 recognition. In both SARS‐CoV‐2 infection mouse models, transfer or combination of COV2‐2196 and COV2‐2130 can maintain the normal weight of mice, reducing viral load and the level of lung inflammation. In addition, as a passive monotherapy transfer, COV2‐2196 protected monkeys against SARS‐CoV‐2 infection. Overall, these results indicated that neutralizing monoclonal antibodies and their combinations thereof, was expected to become a promising prevention or treatment of COVID‐19.^[^ ^23^ ^]^

AZD7442 is a combination of two long‐acting antibodies (COV2‐2196, COV2‐2130). Discovered by Vanderbilt University Medical Center and licensed to AstraZeneca in June 2020, the antibodies have the property of half‐life extension and reduced Fc receptor binding after being optimized by AstraZeneca. LAABs mimic natural antibodies and are being tested clinically for their potential to prevent or treat diseases caused by SARS‐CoV‐2. The trial (NCT04507256) will improve the safety, tolerability as well as pharmacokinetics of AZD7442. It has dosed the first participants with AZD7442 in a Phase I trial. Recently, it has been reported that AZD7442 will move into Phase III clinical trials with over 6000 participants in and outside the United States. One trial will enroll approximately 6000 participants to test the safety and efficacy of AZD7442 to prevent infection, and another trial will evaluate the treatment of AZD7442 for COVID‐19 with approximately 4,000 patients. This combination of antibodies, coupled with half‐life extension technology, can not only reduce the likelihood of viral resistance, but also improve the effectiveness and durability of use.

### 2.5 LY‐CoV016 and LY‐CoV555

LY‐CoV016 (also JS016), is a recombinant fully human anti‐novel Coronavirus monoclonal antibody (JS016) jointly developed by Junshi Biosciences and the Institute of Microbiology of the Chinese Academy of Sciences, etc. A phase I clinical trial (NCT04441918) of LY‐CoV016 has been completed. And the results showed a good toleration of LY‐CoV016 without any drug‐related severe adverse event (SAE).

LY‐CoV555 is a highly active monoclonal antibody isolated from blood samples of COVID‐recovered patients by Eli Lilly. Centered on hospitalized COVID‐19 patients, Lilly initiated a Phase 1 study on LY‐CoV555 and had completed enrolling patients and assessing its safety primarily (NCT04411628) on August 26, 2020. The Phase 1 study was followed by a long‐term follow‐up. Phase I clinical trial results showed that all doses of antibodies are well tolerated without any drug‐related serious adverse event (SAE). A Phase 2 study on COVID‐19 patients in the ambulatory setting (BLAZE‐1, NCT04427501) is being carried out. The latest interim analysis of BLAZE‐1 clinical trial suggested that on day 11, patients in the 2800 mg dose group had a lower viral load than those in the placebo group, while the 700 mg and 7000 mg dose groups failed to meet the primary endpoint. On day 29, the percentage of hospitalized patients in the LY‐COV555 group was 1.6% compared with 6.3% in the placebo group, suggesting that LY‐COV555 reduced the risk of hospitalization and emergency room visits by 75% in patients with mild to moderate COVID‐19.^[^ ^24^ ^]^ Lilly also initiated a Phase 3 study (BLAZE‐2, NCT04497987) on preventing COVID‐19 for patients in long‐term care facilities. Other studies of LY‐CoV555 are in progress, including ACTIV‐2 (NCT04518410), a study sponsored by the National Institutes of Health's on COVID‐19 patients with mild to moderate symptoms. In addition, ACTIV‐3 (NCT04501978) serves as the sole study testing the efficacy of LY‐CoV555 on hospitalized COVID‐19 patients. According to the updated dataset of the trial reviewed on October 26, Data and Safety Monitoring Board (DSMB) has recommended to pause enrollment of ACTIV‐3, as LY‐CoV555 seems to have little effect on advanced COVID‐19 hospitalized patients. There were not significant differences in safety between the groups in this updated dataset. And there was not sufficient evidence to demonstrate that LY‐CoV555 could improve clinical outcomes when provided as combined treatment for hospitalized COVID‐19 patients. Based on data from Lilly's previous study, researchers are still confident that LY‐CoV555 therapy could be used as a safe and effective treatment or preventive agent to benefit patients with mild and moderate COVID‐19.

## 3 NEUTRALIZING SARS‐CoV‐2 THROUGH A NON‐RECEPTOR ANTAGONISM MECHANISM

### 3.1 47D11

Wang et al. first described several neutralizing antibodies generated from hybridomas.^[^ ^25^ ^]^ Among them, a fully humanized antibody named 47D11 shows crosses neutralizing ability to SARS‐CoV and SARS‐CoV‐2. It can be explained by its similar affinity to the S1b domain of both viruses. 47D11 may bind a core domain that is conserved in both viruses but not the epitope in receptor binding interface, which results in its disability to interference receptor binding. The author indicated that 47D11 may play a role in neutralizing SARS‐CoV‐2 through a non‐receptor antagonism mechanism. 47D11 is the first reported human monoclonal antibody that binds the common epitope of SARS‐CoV‐2 and SARS‐CoV and offers the possibility of treating and preventing COVID‐19.

### 3.2 S309

Pinto et al. separated memory B cells from a SARS‐CoV survivor and characterized a series of monoclonal antibodies. S309 is found to strongly neutralize SARS‐CoV and SARS‐CoV‐2. S309 targets a glycan‐containing epitope on the S protein RBD in its open and closed S states, rather than receptor‐binding motif.^[^ ^26^ ^]^ Structural data indicate that 17 of the 22 residues in the S309 identification epitopes are mutual in SARS‐CoV and SARS‐CoV‐2, which explains its cross‐neutralizing ability. Moreover, because S309 can recognize the highly conserved epitopes of N343 glycan in the S1b domain, S309 may have extensive neutralization activity against viruses. When combined with weakly neutral monoclonal antibodies, S309 can show enhanced neutralization and recruit effecting mechanisms. Furthermore, antibody cocktails containing S309 further enhanced the neutralization of SARS‐CoV‐2 and could reduce the appearance of neutralization escape mutants which may reduce the risk of immune escape. Consequently, it provides a method that may be more effective than single antibody therapy.

Vir Biotechnology and GSK, based on S309, generated an antibody drug called Vir‐7831, enhanced its pharmacokinetic characteristics and prolonged the half‐life period while its strong neutralization capacity is obtained. Vir‐7831 is in a randomized, multi‐center, double‐blind, and placebo‐controlled phase 2/3 study (NCT04545060) to assess its safety and efficacy for COVID‐19 patients with mild or moderate symptoms who are at high risk of progression. The primary outcome is the proportion of participants who have progression of COVID‐19 through Day 29. The secondary outcome includes the occurrence of adverse events and incidence and titers of serum ADA to VIR‐7831 and so on. The COMET clinical plan for VIR‐7831 also has another two planned phase I trials to treat severely ill hospitalized patients and to prevent symptomatic infections.

### 3.3 4A8

4A8 can bind to the NTD in each S protein monomer and inhibit both pseudotyped and authentic SARS‐CoV‐2 infection in vitro. No difference is found in the interaction interface. The binding of antibodies does not affect the RBD or the binding of RBD to cell surface receptor ACE2 in space. The recognition epitopes of 4A8 and MARS NTD antibody 7D10 were partially overlapped. The light chain of 7D10 which is close to RBD inhibited the binding of the virus to the DPP4 receptor and inhibited the conformational changes of S protein before and after the RBD‐DPP4 binding (Figure 2). The researchers speculated that 4A8 inhibited virus infection by limiting the conformational change of S protein because its light chain is far away from RBD. Also, the authors found that most isolated monoclonal antibodies could not recognize RBD, and all monoclonal antibodies could not prevent S protein of live SARS‐CoV‐2 from binding to ACE2. It was certainly not anticipated so that in addition to inhibiting virus‐receptor interactions, there are other important mechanisms to neutralize SARS‐CoV‐2.^[^ ^27^ ^]^

## 4 DISCUSSION

Develop neutralizing antibodies against SARS‐CoV‐2 spike protein is a promising approach to combat the COVID‐19 pandemic. Convalescent plasma (CP) therapy is urgently approved as an emergency treatment until an effective antibody drug is developed. Based on limited scientific data, COVID‐19 patients who have undergone convalescent plasma transfusion treatment seem to be safe, improved, and symptoms reduced. However, the efficacy of CP in the treatment of COVID‐19 is controversial. An RCT meta‐analysis showed that the incidence of CP adverse events is low. For children, the elderly, pregnant, tumor, and immunocompromised patients, CP may be a well‐tolerated treatment if the disease cannot be controlled and continues to progress.^[^ ^28^ ^]^ A systematic review identified very low‐certainty evidence on the safety and effectiveness of convalescent plasma for people with COVID‐19.^[^ ^29^ ^]^ In the results of the RCT and the three control NRSIs, there is insufficient evidence to determine whether the convalescent plasma affects the risk of death due to any cause at hospital discharge, time to death, or need for breathing support. Among the 16 non‐controlled NRSIs (5201 participants), some serious adverse effects were found, which could be related to restorative plasma, including death, allergic reactions, or respiratory complications, so it is very uncertain whether or not convalescent plasma affects the number of serious adverse events. In severe COVID‐19 cases, a study showed CP therapy was well tolerated and could improve the clinical outcomes by neutralizing viremia.^[^ ^14^ ^]^ Another study showed that in patients with severe or life‐threatening COVID‐19, convalescent plasma therapy added to standard treatment, compared with standard treatment alone, there was no significant statistical improvement in time to clinical improvement within 28 days.^[^ ^30^ ^]^

In order to make better use of convalescent plasma therapy, it would be important to understand the time, kinetics, and persistence of SARS‐CoV‐2 neutralizing antibodies (NAbs), and their relationship with the severity of clinical disease. Neutralizing antibodies are therapies developed against the coronavirus and have very high specificity. Generally, neutralizing antibodies can treat COVID‐19 patients by eliminating the infectivity of SARS‐CoV‐2, and have dual effects of prevention and treatment. Existing studies that mostly use animal models such as mice, rhesus, etc. There are some differences between the simulation of the infection process of animal models and the process of human infection with viruses. The evaluation index settings for the treatment effect of disease models mostly focus on virus load, alveolar inflammation level, etc., so it is still necessary to establish more suitable, accurate, and comprehensive evaluation indexes.

In addition, antibody therapy currently faces many challenges. Recent data indicate that the specific antibody titer of SARS‐CoV‐2 in severely ill patients increases in the early stage of the disease, while only low levels of neutralizing antibodies are produced in COVID‐19 convalescent. Suggesting that neutralizing antibodies may have a relatively limited effect on controlling the disease. In animal studies, passive immunization with neutralizing antibodies has resulted in remission of the disease, suggesting that neutralizing antibodies may be more important in preventing infection than in solving the disease.^[^ ^31^ ^]^ Besides, the escape mutation of the virus that causes the neutralizing antibody to fail is one main challenge. To address this problem, a possible solution is to design antibodies targeting conserved epitopes in RBD with broad neutralizing properties, which are unlikely to escape from. A combined therapy involving several neutralizing antibodies is another way to combat potential escape mutants. Antibodies in cocktails bind to the key receptors of the spike protein in a non‐competitive manner, which can reduce the ability of mutant viruses to evade single antibody treatment. Broadly neutralizing antibodies such as 47D11 and S309 prevent the virus from infecting cells in other ways without preventing viral S protein from binding to human ACE2 receptors. In addition to using traditional antibody therapy to fight the virus, Sedokani et al.^[^ ^32^ ^]^ imagined using plasmapheresis to remove large numbers of cytokines, low‐affinity antibodies, and antibodies that may mediate antibody‐dependent enhancement (ADE) in the plasma, thereby terminating virus proliferation. Then anti‐ACE2 antibody was used to block the virus from invading tissue cells, and anti‐FcγRII antibody was used to avoid invading the immune system through the ADE‐FcγRII pathway. However, the article also pointed out that plasmapheresis may make patients more susceptible to other pathogenic factors. In addition, antagonizing ACE2 will pose a certain risk, and its antibodies cannot target the enzymatic purpose of ACE2. Therefore, the authors recommend that patients who are suffering from acute respiratory distress syndrome (ARDS) and have no significant response to typical antiviral, and conservative treatments, and those despite receiving adequate treatments, patients whose disease persists or progress can try this therapy. As there is no report about SARS‐CoV‐2 mediated ADE effect, and antagonizing ACE2 may bring huge risks, this treatment plan still needs further discussion and improvement.

Lei et al. synthesized a recombinant protein by linking the ectodomain of human ACE2 with the Fc region of human immunoglobulin IgG1.^[^ ^33^ ^]^ In this study, an ACE2 mutant provides a fusion protein that has low catalytic activity. The recombinant protein shows a high binding affinity to the RBD of SARS‐CoV and SARS‐CoV‐2, and its protective effect on mice was confirmed. The researchers also found that SARS‐CoV and SARS‐CoV‐2 could be cross‐neutralized by the two proteins. This recombinant protein shows great potential in treating COVID‐19 and should be paid further attention.

Soluble ACE2 or ACE2‐Fc protein and its variants have been found to inhibit SARS‐CoV‐2 infection in multiple studies.^[^ ^34^ ^]^ Some of these published/pre‐print studies have shown that ACE2‐Fc could be improved through multiple ways, including introducing mutations to residues at the binding interface, using the human ACE2 ectodomain that keeps its CLD domain, and adopting a tetrameric ACE2 configuration. Currently, a recombinant human ACE2 (APN‐01) has entered phase II clinical trials (NCT04335136), while ACE2‐Fc protein is still in the pre‐clinical stage. Clinical trials of a combination of the recombinant ACE2 or ACE2‐Fc protein and antibody drugs are also urgently needed.

**FIGURE 3:**
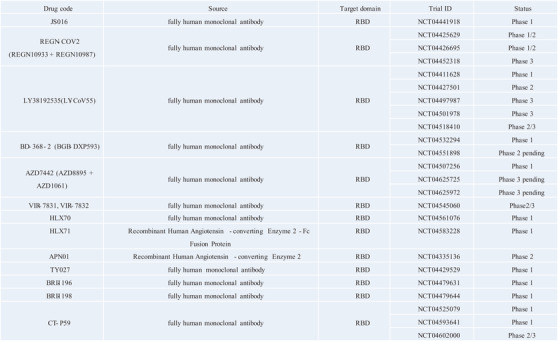
Clinical studies evaluating anti‐SARS‐CoV‐2 monoclonal antibodies or therapeutic antibodies for COVID‐19 under clinical trials

With the deepening of research, the mechanism of action and clinical effectiveness of broadly neutralizing antibodies will be further verified. Moreover, antibodies can either eliminate or enhance viral infection. Although no studies have reported the existence of ADE, the hyperinflammatory response in COVID‐19 and the excess pro‐inflammatory cytokines still need to be considered. Neutralizing antibody therapy has been applied to mild patients in the early stage, and has achieved ideal results. For severe patients, strong neutralizing monoclonal antibodies alone or a combination of different antibodies, or combined with other treatment methods remain to be further studied. SARS coronavirus is widespread and has caused three transmissions in society. Therefore, the development of broadly neutralizing antibodies, the search for more conservative sites, or the use of multiple antibody combination therapies, that is, cocktail therapy, still requires more studies. Some authors have reviewed the research progress of SARS‐CoV‐2 antibodies before us.^[^ ^35^ ^]^ This article supplements the latest virus‐neutralizing antibody progress, supplements and updates their clinical trial progress (Figure 3). At the same time, we discussed some other antibody therapies such as convalescent plasma immunotherapy, combined anti‐ACE2 and anti‐FcγRII therapy, ACE2‐Ig fusion protein therapy, etc. The field of antibody therapy for the COVID‐19 is constantly being updated and progressed rapidly. Many authors and research teams are still required to make continuous efforts and exploration to obtain greater progress to solve the urgent needs of international medical care.

## CONFLICT OF INTEREST

The authors declare no conflict of interest.
